# UDP-glycosyltransferase genes and their association and mutations associated with pyrethroid resistance in *Anopheles sinensis* (Diptera: Culicidae)

**DOI:** 10.1186/s12936-019-2705-2

**Published:** 2019-03-07

**Authors:** Yong Zhou, Wen-Bo Fu, Feng-Ling Si, Zhen-Tian Yan, Yu-Juan Zhang, Qi-Yi He, Bin Chen

**Affiliations:** 1 School of Life Sciences, Chongqing University, Chongqing, 401331 China; 2 Chongqing Key Laboratory of Vector Insects, Institute of Entomology and Molecular Biology, Chongqing Normal University, Chongqing, 401331 China

**Keywords:** *Anopheles sinensis*, UDP-glycosyltransferase (UGT), Diversity and evolution, Pyrethroid resistance, Expression profile, Single nucleotide polymorphisms

## Abstract

**Background:**

UDP-glycosyltransferase (UGT) is an important biotransformation superfamily of enzymes. They catalyze the transfer of glycosyl residues from activated nucleotide sugars to acceptor hydrophobic molecules, and function in several physiological processes, including detoxification, olfaction, cuticle formation, pigmentation. The diversity, classification, scaffold location, characteristics, phylogenetics, and evolution of the superfamily of genes at whole genome level, and their association and mutations associated with pyrethroid resistance are still little known.

**Methods:**

The present study identified UGT genes in *Anopheles sinensis* genome, classified UGT genes in *An. sinensis*, *Anopheles gambiae*, *Aedes aegypti* and *Drosophila melanogaster* genomes, and analysed the scaffold location, characteristics, phylogenetics, and evolution of *An. sinensis* UGT genes using bioinformatics methods. The present study also identified the UGTs associated with pyrethroid resistance using three field pyrethroid-resistant populations with RNA-seq and RT-qPCR, and the mutations associated with pyrethroid resistance with genome re-sequencing in *An. sinensis*.

**Results:**

There are 30 putative UGTs in *An. sinensis* genome, which are classified into 12 families (UGT301, UGT302, UGT306, UGT308, UGT309, UGT310, UGT313, UGT314, UGT315, UGT36, UGT49, UGT50) and further into 23 sub-families. The UGT308 is significantly expanded in gene number compared with other families. A total of 119 UGTs from *An. sinensis*, *An. gambiae*, *Aedes aegypti* and *Drosophila melanogaster* genomes are classified into 19 families, of which seven are specific for three mosquito species and seven are specific for *Drosophila melanogaster*. The UGT308 and UGT302 are proposed to main families involved in pyrethroid resistance. The *AsUGT308D3* is proposed to be the essential UGT gene for the participation in biotransformation in pyrethroid detoxification process, which is possibly regulated by eight SNPs in its 3′ flanking region. The *UGT302A3* is also associated with pyrethroid resistance, and four amino acid mutations in its coding sequences might enhance its catalytic activity and further result in higher insecticide resistance.

**Conclusions:**

This study provides the diversity, phylogenetics and evolution of UGT genes, and potential UGT members and mutations involved in pyrethroid resistance in *An. sinensis*, and lays an important basis for the better understanding and further research on UGT function in defense against insecticide stress.

**Electronic supplementary material:**

The online version of this article (10.1186/s12936-019-2705-2) contains supplementary material, which is available to authorized users.

## Background

UDP-glycosyltransferase (UGT) is a superfamily of enzymes that catalyze glucosidation and help to transfer glycosyl from UDP-glycosyl donator to a variety of lipophilic chemicals. Members of the superfamily share a conserved domain of about 50 amino acid residues located in their C-terminal section, which is believed to contain a binding site for UDP-glycosyl donator. The N-terminal half of the protein exhibits greater sequence divergence between isoforms, providing a binding site for the structurally diverse lipophilic molecules [1–3]. The UGTs play a vital role in the biotransformation of exogenous and endogenous compounds from hydrophobic to hydrophilic, resulting in more efficient excretion to prevent toxic foreign compounds and regulate internal molecules [1, 2, 4]. UGTs’ activities have been documented to be implicated in insect resistance to plant allelochemicals in some insect species [5, 6], especially in Lepidopteran insects, such as the tobacco hornworm *Manduca sexta* [7], the silkworm *Bombyx mori* [8], the Asian corn borer *Ostrinia furnacalis* [9], three *Helicoverpa* species [10], the cotton bollworm *Helicoverpa armigera*, and the tobacco budworm *Heliothis virescens* [11]. The activities were detected mainly in the fat body, midgut and Malpighian tubules [10, 12, 13]. UGTs were also detected in the antenna of insects, and are considered to be involved in olfactory [14, 15]. Additionally, UGTs are involved in some other physiological processes, including cuticle formation [16] and pigmentation [17].

UGTs exist widely in living organisms from bacteria to fungi, plants and animals [1, 2, 4]. With the development of sequencing technique, genome-wide identification of the UGT genes in some insects has been employed to reveal phylogenetics, evolution, expression patterns, and the genes responsible for insecticide resistance. Up to now, whole-genome investigation of UGT genes has been conducted in various insects, most clustered in Lepidoptera and Diptera insects. For example, 33 UGT genes were identified in *Drosophila melanogaster*, and they were classified to five major groups [3, 18]. A total of 42 UGTs clustered in five groups were identified in *Bombyx mori*, 22 UGTs in *Apis mellifera*, and 12 UGTs in *Anopheles gambiae*, respectively [12]. Over 310 putative UGT genes identified from 9 insect species were comparative analysed and classified to families [13], and the nomenclature of UGT sequences was unified for the first time according to the current UGT nomenclature guidelines [19]. Subsequently, 21 UGTs [20], 32 UGTs [21], 20 UGTs [22] were identified in *Plutella xylostella*, *Spodoptera exigua* and *Holotrichia parallela*, respectively. In addition, some researches were reported for the UGT expression in antenna in some species, such as 11 UGTs in *Spodoptera littoralis* [15], and 23 in *Athetis lepigone* [23].

Insecticide resistance has become a threat to agriculture production and vector-borne disease control. UGTs together with the other three major detoxification enzyme families: cytochrome P450 monooxygenases (P450s), esterases (CCEs) and glutathione-*S*-transferases (GSTs), are believed to be involved in insecticide resistance [24, 25]. Members of UGTs often glycosylate the products of Phase I reactions to aid the export of compound from insecticide metabolism, and act as the major phase II enzymes in the detoxification system evolved in all kingdoms of organisms [24, 26, 27]. Some investigations on insecticide resistance have speculated that insect UGTs can mediate the biotransformation of certain toxic xenobiotics, such as organophosphorus [28, 29] and not organophosphorus [30–35]. UGT46A6 was suggested to play a role in detoxification due to its up-regulation by topical application of pyrethroid on the antennae [36], and toxicology research proved that UGT2B17 was involved in chlorantraniliprole resistance [37]. The information indicates that glycoside conjugation mediated by UGTs play an important role in insecticide detoxification.

The mosquito *Anopheles sinensis*, as the major vector of vivax malaria and *Brugia malayi* filariasis in Southeast Asia [38], has high abundance and increasing vectorial capacity. It is usually controlled by use of pyrethroids for indoor spraying and insecticide-impregnated bed nets [39]. However, extensive and continued application has led *An. sinensis* to evolve a high insecticide resistance [40]. Target-site insensitivity, as one of predominant mechanisms, has been reported in this mosquito species [41–43]. Recently, genome sequencing in *An. sinensis* facilitated identification and characterization of some families of genes involved in pyrethroid resistance, such as P450s [44, 45], GSTs [44], CCEs [44, 46], cuticular proteins [47], ionotropic glutamate receptors [48] and odorant-binding proteins [49]. Besides, enzyme overproduction and enzyme modification caused by coding sequence alteration (another way to enhance the metabolic capability) have also been reported in some insect species [50], but not in UGT genes of *An. sinensis*. To date, the diversity, classification of UGTs and the potential UGT genes involved in insecticide resistance are still quite limited, not only in *An. sinensis* but also in other insects.

The present study identified and analysed all the 30 putative UGTs in *An. sinensis* genome, investigated expression profile of these UGT genes in response of pyrethroid resistance using RNA sequencing (RNA-seq) and reverse-transcription quantitative-PCR (RT-qPCR), and determined the variations in coding sequences (CDs) of these genes in pyrethroid-resistant populations using re-sequencing technique. This is the first comprehensive screening for the UGT genes potentially involved in pyrethroid resistance, and is an important basis for further study on the biology and function of UGTs in insecticide resistance.

## Methods

### Identification of putative UGT genes in *Anopheles sinensis*

Putative UGTs members were detected from the *An. sinensis* genome, which was sequenced by Institute of Entomology and Molecular Biology, Chongqing Normal University (publication in preparation). The assembly of genome covered 98.35% of protein-coding expressed sequence tags (ESTs) generated by transcriptome sequencing [51]. Two sets of transcriptome data of *An. sinensis* [51, 52], downloaded from National Center for Biotechnology Information (NCBI) as the EST database, were also used in the identification of putative UGTs in *An. sinensis*.

Three methods were utilized to ensure comprehensive identification of all putative UGT genes. First, the BLASTP searching against protein database of *An. sinensis* was performed with an E-value cut-off as 1e−5 [53], using UGT amino acid sequences of *An. gambiae* and *Dr. melanogaster* downloaded from NCBI as queries. Second, the Hidden Markov Model (HMM) for UGT family (Pfam number: PF00201) downloaded from Pfam (version 26.0) (http://pfam.sanger.ac.uk/) was used as queries to search against protein database of *An. sinensis*. Third, the TBLASTN was performed with an E-value cut-off as 1e−5 to search for the *An. sinensis* genome, using UGT sequences of *An. sinensis* identified above as queries. The sequences detected by the three approaches were combined as the eventual putative UGTs in *An. sinensis*. Two methods were used to confirm the authenticity of UGT genes. First, the putative UGT sequences were predicted by Smart (http://smart.embl-heidelberg.de/) [54] to determine the conserved UGT domain. Second, all putative sequences were used as queries to search against the previous EST database with an E-value of 1e−10. The full-length of the putative UGT sequences were determined using Fgenesh + (http://www.softberry.com/) with *An. gambiae* as reference. For partial genes, full-length sequences were determined by extending the flanking regions (1 kb or longer), and manually comparing with correspondingly homologous UGT genes.

### Phylogenetic analysis and nomenclature of *Anopheles sinensi*s UGT genes

The amino acid sequences of UGTs in *An. sinensis* (30 UGTs) and the other three Diptera species, *An. gambiae* (23), *Aedes aegypti* (32) and *Drosophila melanogaster* (34) downloaded from NCBI were applied in the phylogenetic analysis. The Clustal X [55] was used for sequence alignment, and the Modeltest 3.7 [56] was used for the selection of best-fit nucleotide substitution model. The phylogenetic relationships of UGTs were constructed by maximum likelihood (ML) using MEGA version 6.0 [57]. The bootstrap values for 1000 replicates were calculated and marked on branches of resulted phylogenetic tree. The tree was modified by an online tool, Interactive Tree of Life (http://itol.embl.de/) [58] and decorated in Adobe Photoshop^®^ CS6.

Names of *An. sinensis* UGTs were preliminarily assigned in reference of the orthologue with those of other three Diptera species [13] based on the phylogenetics analysis. The names were then adjusted according to the nomenclature guidelines of the UGT Nomenclature Committee [19]. The UGTs classification of family was determined with 40% or greater amino acid sequence identity (aaID) as a threshold, and the UGTs classification of sub-family was further determined with sequences of 60% or greater amino acid sequence identity (aaID). Sequence identity was computed using BLASTP [53].

### The expression profile of UGT genes in *Anopheles sinensis* pyrethroid-resistant populations

RNA sequencing (RNA-seq) of four mosquito populations/strain were conducted using Illumina HiSeq™ 2000 (Illumina, San Diego, CA, USA) at Beijing Genomics Institute (BGI). These four populations/strain include three field pyrethroid-resistant populations, which were collected from Anhui (AH), Chongqing (CQ), Yunnan (YN) provinces, and one laboratory pyrethroid-susceptible strain which was originated from Wuxi, Jiangsu province. For three field-resistant populations, mosquito larvae or pupae collected from rice (*Oryza sativa*) fields were locally reared to adults. Female *An. sinensis* adults 3-days post emergence were tested for pyrethroid susceptibility using the standard World Health Organization (WHO) tube bioassay with pyrethroid of 0.05% concentration on test papers [59]. The mosquitoes of laboratory susceptible strain were prepared using the same process. Total RNA was extracted from a pool of 15 mosquitoes for each RNA-seq sample using Trizol Reagent (Invitrogen) according to the manufacturer’s protocol.

Reads obtained from sequencing were cleaned by removing reads with adaptor, or with N > 10% (N: A/T/G/C) or with low quality (exceeding 50% nucleotide of quality value ≤ 10) using in-house perl script, and mapped to the *An. sinensis* genome sequenced by Institute of Entomology and Molecular Biology, Chongqing Normal University (publication in preparation) using TopHat [60]. The expression levels were determined in terms of fragment per kb per million reads (FPKM) using Cufflinks [61]. Differential accumulation of transcripts between pyrethroid-resistant and -susceptible mosquitoes was assessed by the Cuffdiff program within Cufflinks. To minimize the impact of sequencing length and nucleotide composition, FPKM for each gene of each sample were calculated to determine the expression quantity [62]. The normalized gene expression level of *An. sinensis* UGT genes in the three field-resistant populations (AH-FR, CQ-FR, YN-FR) was each compared with the laboratory susceptible strain (WX-LS). Genes with FPKM log_2_(fold) ≥ 1 and ≤ − 1, and with *P*-value ≤ 0.05 were considered to be differentially up- and down-expressed, respectively.

### RT-qPCR verification of UGT genes associated with pyrethroid resistance

Seven UGT genes significantly up-/down-regulated in at least two resistant populations were subject to RT-qPCR analysis to validate the expression results from RNA-seq analysis. The three pyrethroid-resistant populations and one laboratory-susceptible strain, same as in RNA-seq analysis, were applied in the RT-qPCR for the expression verification. The gene-specific primers for RT-qPCR amplification were designed using Primer Premier 5.0 (Premier Biosoft International, Palo Alto, CA, USA) (Table 1). Total RNA extraction was performed using Trizol Reagent (Invitrogen) according to the manufacturer’s instructions. Complementary DNAs (cDNAs) were synthesized from 1.0 μg RNA using PrimScript TM RT Reagent Kit with gDNA Eraser (TaKaRa, Dalian, China) and stored at − 20 °C.

**Table 1:** Primers used in RT-qPCR verification for seven UGT genes

Gene name	Forward prime (5′–3′)	Reverse prime (5′–3′)	Product size (bp)
*UGT308D3*	TTAAGCCCAAGCCACTACCG	GGTAGAGCCTCCATCGCATC	154
*UGT308C3*	GTTTCGGTTTGACCTGGTGC	GATCACCTCCGTCGAGTACG	128
*UGT308F2*	ACCACACTTTACGCAGCTCA	ATTGCGGTCAGTTCCGGTAG	132
*UGT308G2*	GCAAAGCGGCTCACAATTCA	TTGGTCCCTGAACAACCGAG	126
*UGT308G3*	TCCGATCAGTCCAAACTGCC	CTCAGAAGTCCACTGTGCGT	121
*UGT308G4*	GCCAAACATGATTCCCGTCG	AACAGATCACTTCGGGCGTT	120
*UGT302A4*	CCAAATGCCAAGCGTTCCTT	CGTGAAACCCTCCAACCTCT	130

Real-time reactions were conducted on a thermal cycler (CFX, Bio-Rad, USA) in a 15 μL total reaction volume containing 7.5 μL of 2× qPCR mix (Bio-Rad, USA), 0.5 μL each of gene-specific primers and 1 μL the cDNAs templates, and 5.5 μL of double distilled water. Thermal cycling conditions were 94 °C for 3 min; 40 cycles of 95 °C for 5 s, 60 °C for 15 s, and 72 °C for 15 s, and followed by a dissociation analysis to check the homogeneity of the PCR product. All RT-qPCRs were conducted with three biological replicates (three mosquitoes per sample) and three technique replicates, each technique replicate with a new preparation of RNA sample.

The relative expression levels of each gene were normalized to two genes (the ribosomal protein S7 (RPS7) and ribosomal protein L49 (RPL49)) using the 2^−∆∆Ct^ method. The statistical significance of the gene expression was calculated using a Student’s t test for all 2-sample comparisons and one-way analysis of variance (ANOVA) for multiple sample comparisons (SAS v9.1 software). *P*-value < 0.05 was considered to be statistically significant.

### Variant screening of UGT genes in *Anopheles sinensis* resistant populations

A total 36 individuals were re-sequenced using Illumina HiSeq™ 2000 (Illumina, San Diego, CA, USA) at Beijing Genomics Institute (BGI). These individuals included six pyrethroid-resistant ones from each field-resistant population (AH-FR, CQ-FR, YN-FR) and six pyrethroid-susceptible ones from each field-susceptible population (AH-FS, CQ-FS, YN-FS). The field samples collected were preserved in 85% alcohol, one leg of each mosquito was separated for species molecular identification using the amplification-specific ITS2 and 28S rDNA [63] with the Fast Tissue-to-PCR Kit (Fermentas) and the remaining mosquito body was used for genomic DNA extracting using QIAGEN DNeasy Blood and Tissue Kit (Duesseldorf, German) and re-sequencing. Paired-end sequencing libraries with insert sizes of 500 bp were constructed according to the manufacturer’s instructions.

Paired-end reads obtained from re-sequencing were cleaned and mapped to the *An. sinensis* genome using BWA software [64] with the parameters “bwa aln -n 0.04 -x 650 -l 35 -R 20 -t 4 -e 30 -i 15” and “bwa sampe -a 500” and other parameters in default. The Genome Analysis Toolkit (GATK, version 2.4-9) with re-alignment algorithm and default parameters was used to identify single nucleotide polymorphisms (SNPs) [65]. The SNPs obtained were further filtered using the parameters, “QD ≥ 2.0, MQ ≥ 40.0, ReadPosRankSum ≥ − 8.0, FS ≤ 60.0, HaplotypeScore ≤ 13.0, MQRankSum ≥ − 12.5”.

Three criteria, allele frequency-based filtering, Fisher’s exact test and *F*_ST_, were applied to identify differential SNPs associated with pyrethroid resistance in the three resistant populations in comparison with corresponding susceptible populations. Firstly, non-synonymous SNPs in CDs were screened in three field-resistant/susceptible population pairs, and those that met all three criteria were considered to be differential SNPs potentially leading to enzyme modification: [f(FR) − f(FS) ≥ 50%] (metric being positive or negative), Fisher’s exact test *P*-value ≤ 0.05, and *F*_ST_ value ≥ 0.2 and with *F*_ST_ *P*-value ≤ 0.05. Secondly, SNPs in assumed regulation regions of UGT genes (up-/downstream 5 kb intergenic regions, including untranslated regions in this research) were also screened in three field-resistant populations compared with the laboratory-susceptible strain, and those that met criteria: [f(FR) − f(LS) = 1] (metric being positive or negative) were considered to be differential SNPs potentially impacted in UGT transcription regulation.

## Results

### Diversity of UGT genes in *Anopheles sinensis*

A total of 30 putative UGT genes are identified from *An. sinensis* genome, and they encode 466–570 amino acids (a.a.) (Table 2). Among them, the *UGT50B4* is predicted to be lack of transcriptional start site (TSS) and transcript support; therefore, it is suggested to be a pseudogene. All other UGT genes are supported by transcript but the *UGT308G4* has only partial sequence due to incompleteness in the genome assembly.

**Table 2:** Detailed information of the 30 *Anopheles sinensis* UGT genes

Gene name	Annotation number^a^	Amino acid length	Scaffold	Gene position		Chain	Exon number
*UGT301A3*	–	531	scaffold56	1,652,531	1,654,341	+	4
*UGT301A4*	–	568	scaffold56	1,657,070	1,663,417	+	7
*UGT301C2*	–	516	scaffold56	1,671,094	1,672,725	+	2
*UGT302A3*	As10010026	523	scaffold15	2,726,835	2,728,683	–	5
*UGT302A4*	As10010028	532	scaffold15	2,737,506	2,739,389	–	5
*UGT302H3*	As10010016	527	scaffold15	2,680,439	2,682,233	+	4
*UGT302J2*	As10010015	531	scaffold15	2,676,766	2,678,998	–	2
*UGT306C2*	As10005877	501	scaffold20	834,452	836,043	+	2
*UGT306C3*	As10003223	501	scaffold6	401,713	403,299	+	2
*UGT306D2*	As10005507	519	scaffold46	2,795,691	2,797,393	–	3
*UGT308A3*	As10010708	528	scaffold25	1,159,218	1,160,942	+	3
*UGT308B3*	As10010710	499	scaffold25	1,163,953	1,165,665	+	4
*UGT308C3*	As10010707	518	scaffold25	1,156,090	1,157,927	–	5
*UGT308D2*	As10004236	533	scaffold18	462,698	464,424	+	3
*UGT308D3*	As10004237	528	scaffold18	465,105	466,827	+	3
*UGT308F2*	As10010705	570	scaffold25	1,153,125	1,155,036	+	4
*UGT308G2*	As10007651	519	scaffold51	1,435,858	1,437,480	–	2
*UGT308G3*	As10007652	524	scaffold51	1,440,208	1,441,840	–	2
*UGT308G4*	As10007653	494^b^	scaffold51	1,444,996	1,446,538	–	2^b^
*UGT308H2*	As10010709	495	scaffold25	1,161,285	1,163,015	+	4
*UGT309C1*	As10005308	531	scaffold22	2,366,476	2,369,071	+	3
*UGT310B2*	As10016551	521	scaffold14	30,186,316	30,188,063	+	3
*UGT313B2*	–	540	scaffold56	4,432,004	4,437,360	–	4
*UGT314A3*	As10015698	544	scaffold14	19,089,263	19,094,021	–	4
*UGT315A3*	As10010457	550	scaffold15	7,356,301	7,358,273	–	5
*UGT36B3*	As10006960	525	scaffold1	1,429,881	1,431,575	+	3
*UGT36C3*	As10006957	517	scaffold1	1,412,540	1,416,506	+	4
*UGT49A4*	As10004227	523	scaffold18	375,909	378,827	–	3
*UGT50B4* ^c^	As10015288	429^b^	scaffold14	13,800,532	13,804,587	–	6^b^
*UGT50B5*	As10015287	466	scaffold14	13,780,247	13,799,802	–	7

^a^From gene set produced from the gene annotation of the genome sequencing

^b^Partial sequences, or partially identified exon numbers

^c^Pseudogene

**Fig. 1:**
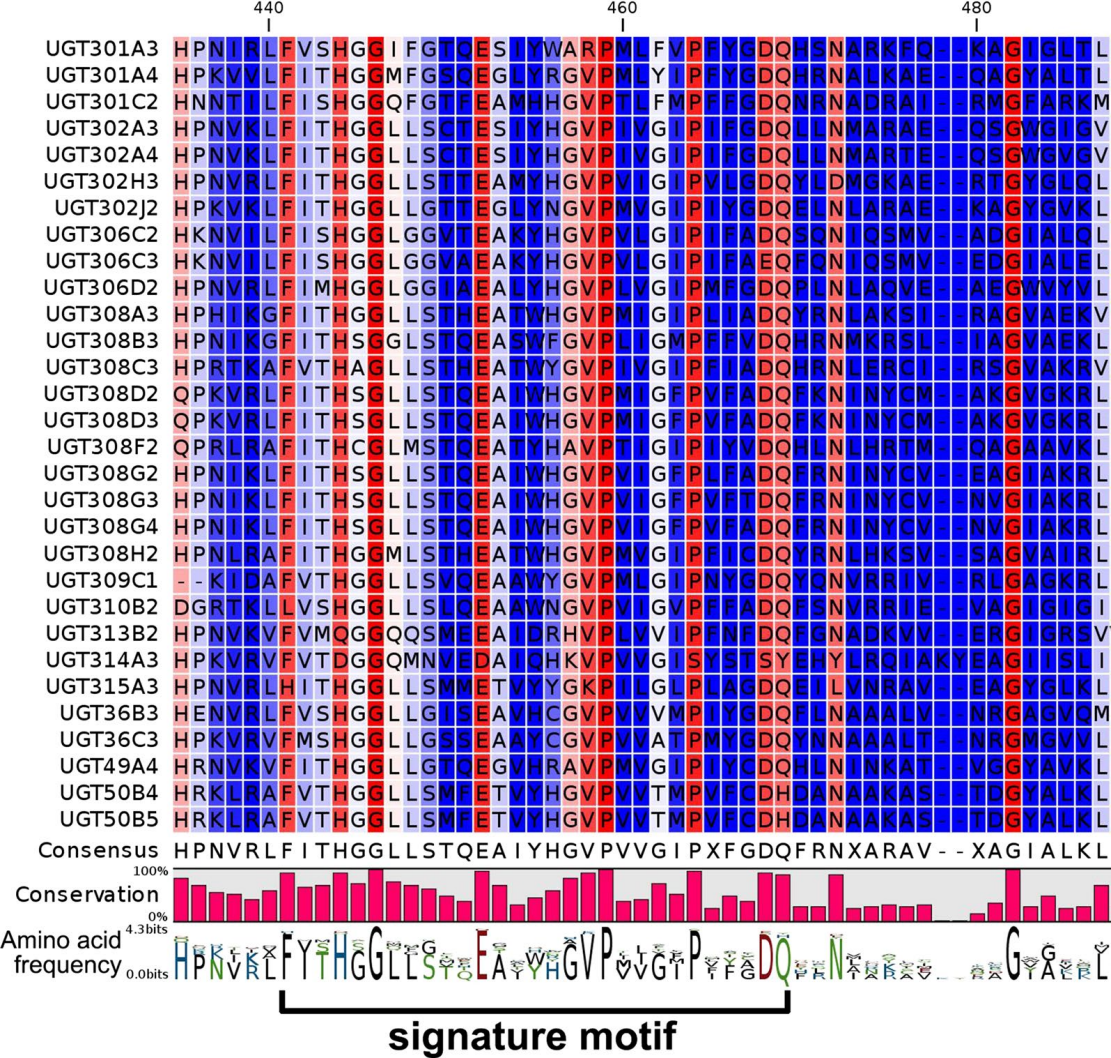
Signature motif of 30 *Anopheles sinensis* UGT amino acid sequences. The conservation of amino acids is described using consensus sequence (X indicted any amino acid), per cent conservation and amino acid frequency beneath the alignment

In the C-terminal domain of UGTs, there is a signature motif sequence that is thought to be involved in the binding of the UDP moiety of the nucleotide sugar, and therefore is considered as a diagnostic characteristic of UGT sequences [19]. In the present research, the multiple alignment of 30 putative *An. sinensis* UGTs reveals the good conservation of signature sequences, which confirms the UGT gene identification (Fig. 1). The signature motifs are 29 a.a. long (consensus sequence: FITHGGLLSTQEAIYHGVPVVGIPXFGDQ, with X indicting varying amino acid). Remarkable homology occurs in the signature motifs with two residues even having 100% conservation (G446 and P459 on the alignment). The conservation of signature motifs was also reported in UGTs of other insects [12, 20]. In addition, multiple alignment of 11 representative members from 30 putative *An. sinensis* UGTs show that the predicted UDP-glucuronic binding regions (donor binding regions, DBR1 and DBR2) are also conserved in *An. sinensis* UGTs compared with other UGTs from mammals or insects [13, 66] (Additional file 1**)**. In detail, the conserved residues in two DBRs include four nucleotide interacting residues (S248, W302, Q305, E335), two phosphate interacting residues (T326 and H327) and two glucoside interacting residues (D351 and Q352). Since C-terminal half is believed to bind the sugar donor [1], all these highly conserved residues in this half might play an important role in sugar binding. A negatively charged amino acid residue (D453) is highly conserved, which is suggested to be involved in positioning and orienting the membrane domain [13] (Additional file 1**)**. In comparison with conserved C-terminal, N-terminals of UGTs are highly variable and lead to the diversity of UGT (Additional file 1**)**. However, two residues (H31 and D93) in N-terminal are found conserved as reported in the previous study, and are considered to play a critical conserved functions in catalytic action since N-terminal is believed to bind acceptor molecules [13]. The N-terminal signal peptides of the 11 representative UGTs are predicted to be 20–36 a.a., which is involved in UGTs’ integration into the endoplasmic reticulum compartment [1] (Additional file 1).

**Fig. 2:**
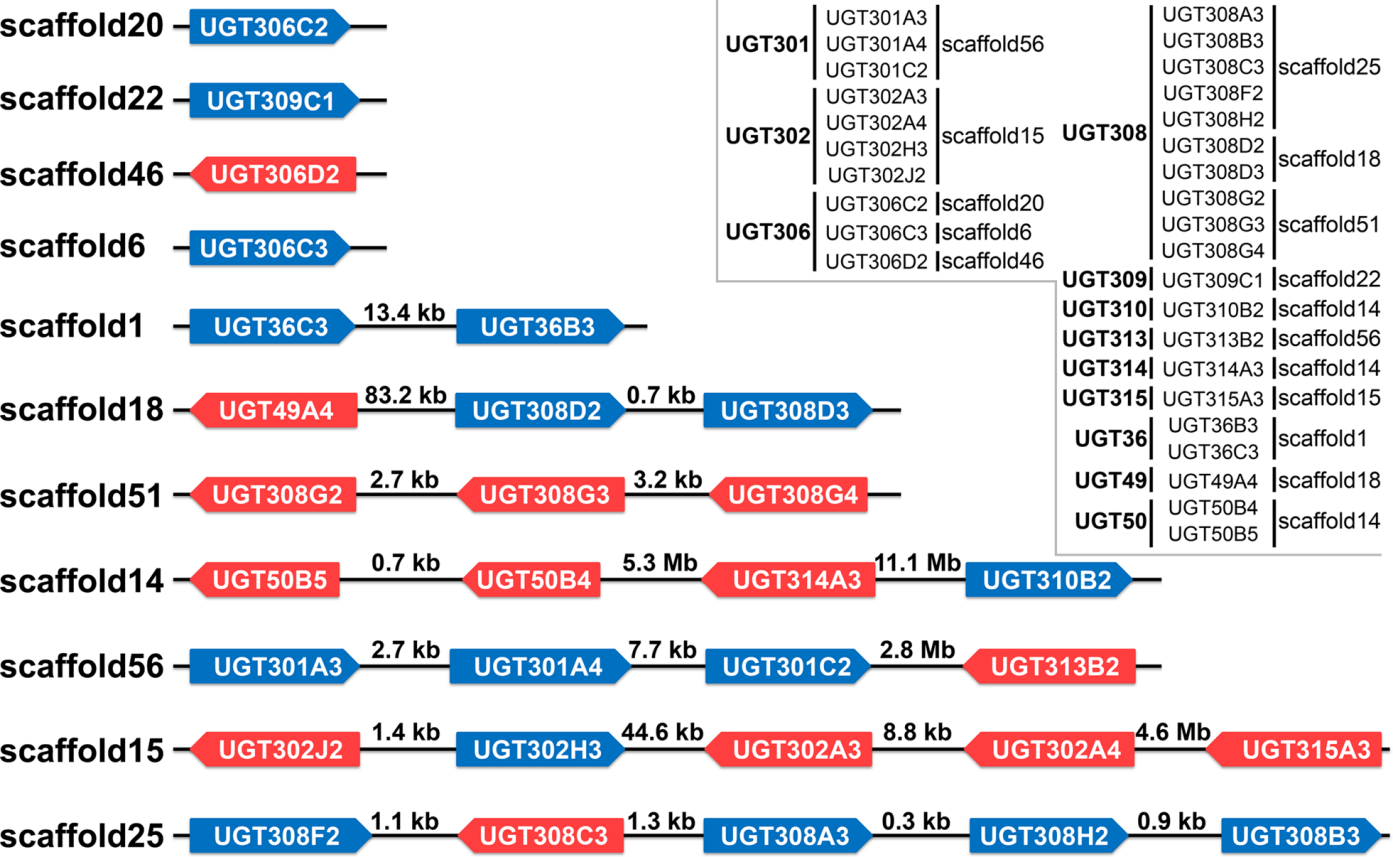
Genomic location of 30 UGT genes in *Anopheles sinensis.* All 30 putative UGT genes identified in *An. sinensis* genome are shown on scaffolds. Colour-filled boxes represent UGT genes with the box length corresponding to relative sequence length, and with the blue and red colours indicating the 5′–3′ and 3′–5′ directions of the sequences, respectively. The horizontal lines represent intergenic regions with the length marked on the lines, and the genes’ classification to families is noted on the slot in upper right

The 30 *An. sinensis* UGT genes are unevenly located on 11 scaffolds, including four scaffolds (scaffold20, 22, 46, 6) with only one gene, the scaffold1 with two genes, scaffold18 and scaffold51 each with three genes, scaffold14 and scaffold56 each with four genes, and scaffold15 and 25 each with five genes (Fig. 2). Among these genes, 16 genes are located in the positive strand of the genome and 14 in the negative chain. According to the nomenclature guidelines of the UGT Nomenclature Committee [19], the 30 UGT genes are classified into 12 families (UGT301, UGT302, UGT306, UGT308, UGT309, UGT310, UGT313, UGT314, UGT315, UGT36, UGT49, UGT50), and further into 23 sub-families (Fig. 2, Table 2). All three genes in UGT301 family, four in UGT302, two in UGT36, and two in UGT50 are present in scaffold56, scaffold15, scaffold1, and scaffold14, respectively, which suggests that these genes in each of these four families be possibly closer in phylogenetics and derived due to gene duplicate events (Fig. 2 slot). For 10 genes in UGT308 family, five, two and three genes are located in scaffold25, scaffold18 and scaffold51, respectively, which suggests that at least part of genes be possibly closer in phylogenetics and derived due to gene duplicate events. Whether the scaffold25, scaffold18 and scaffold51 are adjacent in chromosome, requires further chromosome location analysis. Three genes in UGT306 family are each located in a unique scaffold, and their relationship and origination could not be concluded due to lack of chromosome location information. For the other six families, there is only one gene each, which is separately present in different scaffolds.

### Phylogenetics and evolution of *Anopheles sinensis* UGT genes

**Fig. 3:**
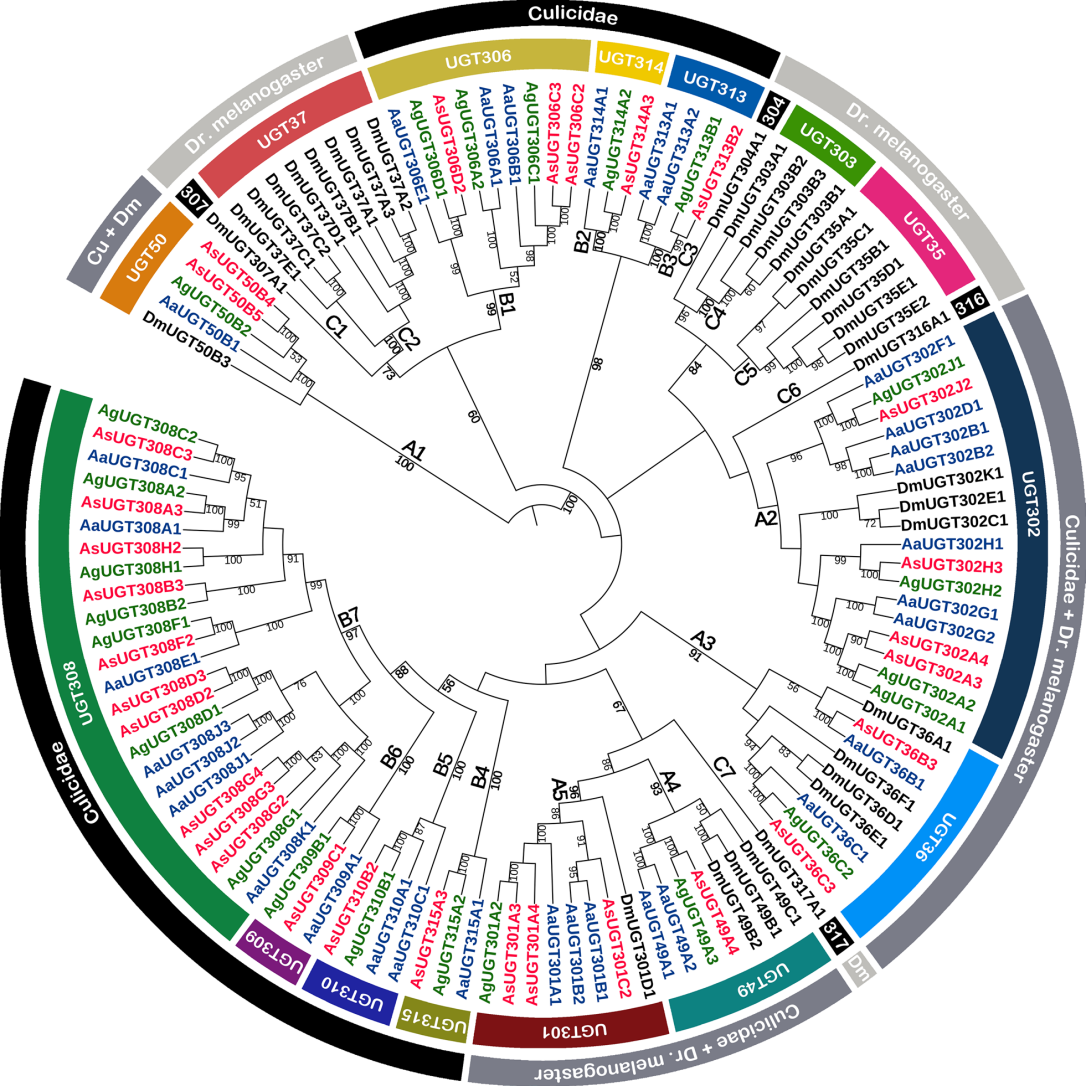
Phylogeny relationships of UGT amino acid sequences in *Anopheles sinensis*, *Anopheles gambiae*, *Aedes aegypti* and *Drosophila melanogaster.* Phylogenetic tree was constructed based on maximum likelihood (ML) using MEGA version 6.0. Bootstrap values (1000 replicates) larger than 50% are marked on corresponding branches. The species belonging and family classification of the UGT genes are marked on the outmost and second outer color-filled circles. As, *An. Sinensis*; Ag, *An. Gambiae*; Aa, *Ae. Aegypti*; Dm, *Dr. melanogaster*; Cu, Culicidae

A total of 119 UGT genes from the four Diptera species, *An. sinensis* (30 genes), *An. gambiae* (23), *Aedes aegypti* (32) and *Drosophila melanogaster* (34), are divided into 19 traditional families according to the previous classification guidelines [19] (Fig. 3). Based on the traditional classification of families, these 19 UGT families shows apparent patterns of interspecific conservation and lineage-specific expansion. Seven families of the 19 families are specific to three Culicidae species (UGT306, UGT313–315 and UGT308–310) while other seven families are species-specific to *Drosophila melanogaster* (UGT307, UGT37, UGT304, UGT303, UGT35, UGT316, UGT317), and the remaining five are common in all four Diptera species investigated (UGT50, UGT302, UGT36, UGT49, UGT301).

Five families (branch A1, A2, A3, A4, A5) are common for the four Diptera species investigated. The UGT50 family (A1), UGT36 (A3), UGT49 (A4) and UGT301 (A5) have the bootstrap value support of 100, 91, 93, and 96%, which suggests that these UGT families be monophyly. The UGT50 family occupies a basal position in the phylogenetic tree, and has one gene for each species except for *An. sinensis* that has two genes (*UGT50B4* and *UGT50B5*). The two genes are closely located on scaffold14 with 71% aaID, which suggests that *An. sinensis* UGT50 genes experienced recent gene duplication event. In the UGT36 family (A3), there are two genes, one, two and four in *An. sinensis*, *An. gambiae*, *Aedes aegypti* and *Drosophila melanogaster*, respectively. The UGT36 genes might earlier evolve in two branches, and the UGT36 genes in *Drosophila melanogaster* might experience gene expansion. The families UGT49 (A4) and UGT301 (A5) group together as sister branches with the bootstrap value support of 86%. The UGT49 family contains only one gene in *An. sinensis*, and appears expanded in *Drosophila melanogaster*, whereas the UGT301 genes in mosquitoes expanded. The family UGT302 (A2) contains four UGTs in each of *An. sinensis* and *An. gambiae*, seven in *Aedes aegypti* and three in *Drosophila melanogaster*. The branch for UGT302 is not well supported by bootstrap value, and the evolutionary relationship among these UGT302 genes could not be well elucidated. Within this family, double orthologous pairs are found across three mosquito species investigated (*UGT302A3*–*4*, *UGT302A1*–*2* and *UGT302G1*–*2* in *An. sinensis,* and *An. gambiae* and *Aedes aegypti*, respectively).

Seven families, UGT306 (B1), UGT314 (B2), UGT313 (B3), UGT315 (B4), UGT310 (B5), UGT309 (B6), and UGT308 (B7) are specific for three mosquito species investigated. The branches for these families are supported by at least 97% of bootstrap values, which suggests these families in traditional classification be monophyly. The UGT306 genes (B1) might earlier evolve into two branches. Two *An. sinensis* UGTs (*UGT306C2* and *UGT306C3*) group together, and are each located in a unique scaffold, and their relationship could not be concluded due to lack of chromosome location information although the two genes share high 93% aaID. The UGT314 (B2) and UGT 313 (B3) families are sister each other in phylogenetics, the branch of these two families are supported by 98% bootstrap value, and they might be combined into one family with further support of research results. The UGT315 (B4), UGT310 (B5) and UGT309 (B6) families each have at most two genes for the three mosquito species, whereas UGT308 family (B7) much expanded with each of mosquito species with at least seven genes. The UGT315 and UGT309 appear to be conserved to three mosquito species, and this suggests that genes in the two families might be separately originated from their ancestor sequences. The UGT308 family is the largest family in the UGT superfamily in terms of gene number, and shows remarkable gene expansion. Close 1:1 orthologous genes between *An. sinensis* and *An. gambiae* are more common in this family; however, there are three more genes in *An. sinensis* in UGT308D and UGT308G. The gene expansion in those two sub-families might have resulted from a recent gene duplication event (the *UGT308D2*–*D3* are tandemly arranged on scaffold18 with 83% aaID; the *UGT308G2*–*G3* are tandemly arranged on scaffold51 with 78–93% aaIDs).

There are also seven families to be specific for *Drosophila melanogaster*, and they are UGT307 (C1), UGT37 (C2), UGT304 (C3), UGT303 (C4), UGT35 (C5), UGT316 (C6), and UGT317 (C7). Most *Drosophila melanogaster* UGTs (22 of 34 UGTs) are classified into these seven specific families, and further 18 of the 22 genes group into three families (UGT35, UGT303, UGT37), which suggests that significant lineage-specific radiations happened in those three families. The phylogenetic relationships of UGT members in these three families are consistent with the previous researches [3, 12, 13]. There is only one gene in UGT307 family (C1) in *Drosophila melanogaster*, and eight genes in UGT37 (C2) with their clade supported by 100% bootstrap value. Whether the families UGT307 and UGT37 (together with 73% bootstrap value support) should be combined into one family needs further research. Similarly, there is one gene in UGT304 (C3), four genes in UGT303 (C4) (supported by 100% bootstrap value), and six genes in UGT35 (C5) (no obvious bootstrap value support). Whether the UGT304 should join UGT303 (together with 96% bootstrap value support), and whether UGT304, UGT303 and UGT35 (together with 84% bootstrap value support) should be combined into one family is worthy of further research. The UGT316 (C6) groups with A2 with no obvious bootstrap value support, while the UGT 317 (C7) occupies a separate position next to the sister branches A4 and A5 (supported by 67% bootstrap value), and both of them have only one gene.

### The relative expression of UGT genes in *Anopheles sinensis* resistant populations

**Fig. 4:**
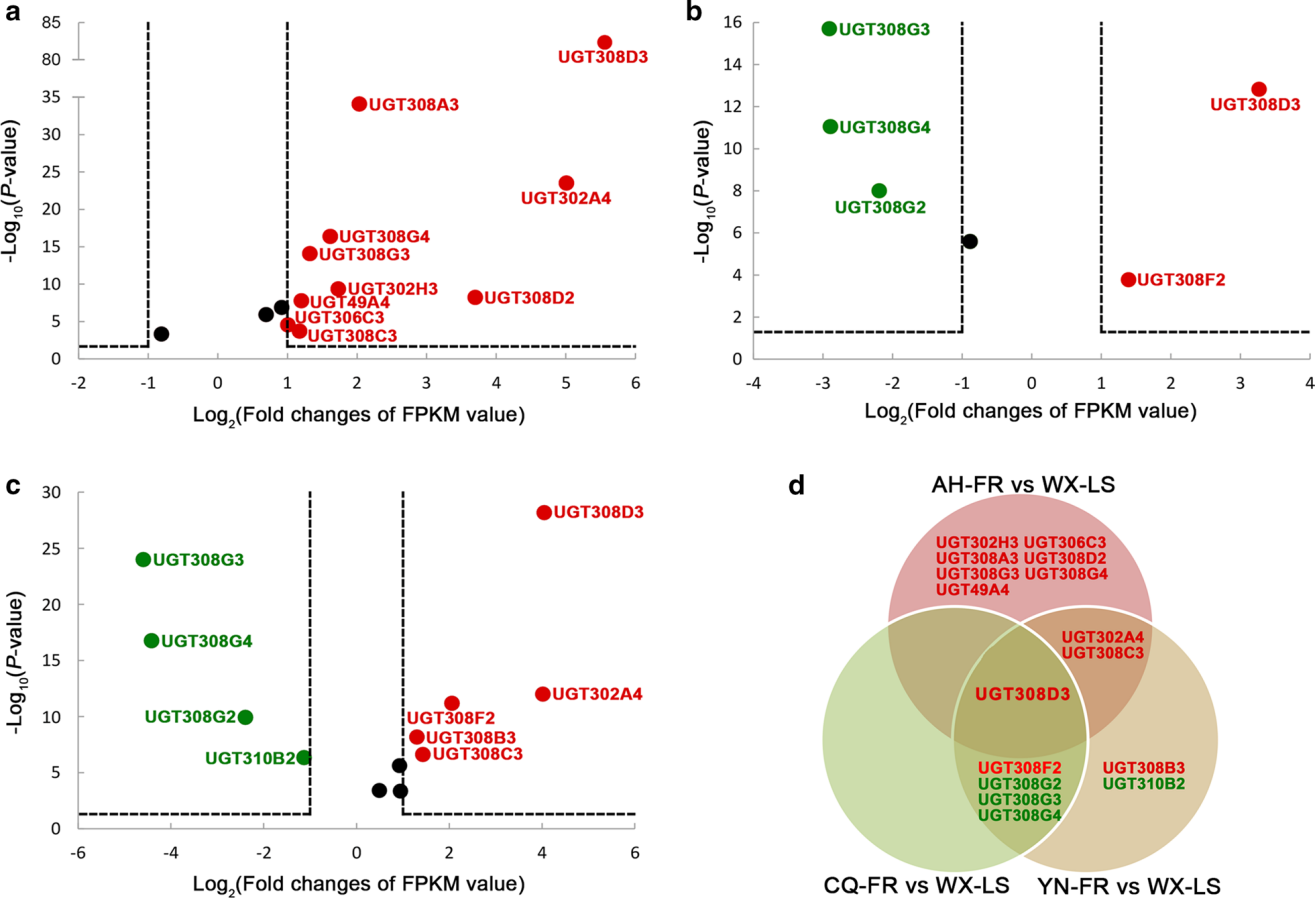
Differential expression of 26 *Anopheles sinensis* UGT genes detected by RNA-seq. **a**–**c** The differential expression in Anhui (AH-FR), Chongqing (CQ-FR) and Yunnan (YN-FR) field pyrethroid-resistant population compared with the laboratory susceptible strain (WX-LS), respectively. Genes with FPKM log_2_(fold) ≥ 1 and *P*-value ≤ 0.05 are considered to be significantly upregulated, and are indicated in red, while those with FPKM log_2_(fold) ≤ − 1 and *P*-value ≤ 0.05 are regarded as significantly downregulated, and are indicated in green. Black-filled cycles represent those genes that have no significant expression. **d** The Venn diagram summarizes the genes with significantly different expression in the three resistant populations, and the gene *UGT308D3* is significantly upregulated in these three populations

Insecticide resistance can be developed by multiple routes [50]. In the present study, RNA-seq analysis was firstly conducted to investigate the UGT members potentially associated with pyrethroid resistance at the transcription level. Compared with the laboratory susceptible strain (WX-LS), 14 UGT genes in five families (nine in UGT308, two in UGT302, and one in each of UGT306, UGT310 and UGT49) are significantly differentially expressed (DEGs) in at least one field pyrethroid-resistant population, with 10 genes upregulated and four downregulated (Fig. 4). Among the 14 DEGs, there are 10 genes in AH-FR (all upregulated) (Fig. 4a), five in CQ-FR (two upregulated and three downregulated) (Fig. 4b) and nine in YN-FR (five upregulated and four downregulated) (Fig. 4c), respectively. The *UGT308D3* gene is significantly upregulated in all three field resistant populations, the *UGT308C3* and *UGT302A4* are significantly upregulated in both AH-FR and YN-FR, and the *UGT308F2* is significantly upregulated in both CQ-FR and YN-FR; however, three genes (*UGT308G2*–*4*) are significantly downregulated in CQ-FR and YN-FR (Fig. 4d). These seven genes are considered to be the main candidates for involvement in pyrethroid resistance. The remaining seven genes significantly up- or downregulated in only one resistant population are considered to be secondary candidates. The expression pattern suggests that the UGT genes involving insecticide resistance differ due to different geographical populations, which resource probably from different genetic backgrounds.

### RT-qPCR verification of seven UGT genes potentially involved in pyrethroid resistance

**Fig. 5:**
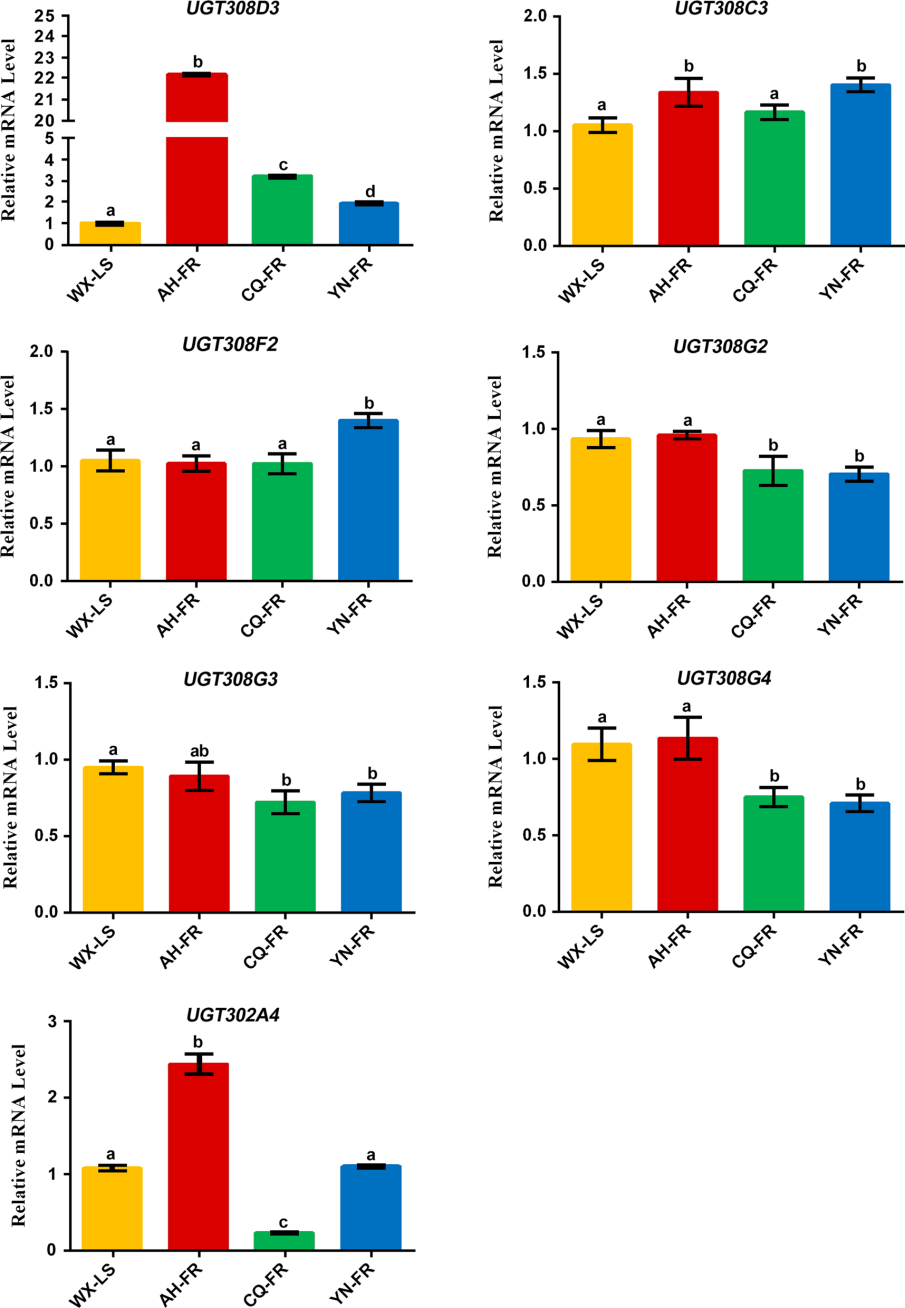
RT-qPCR verification of seven UGT genes. The relative expression levels of three pyrethroid-resistant populations (AH-FR, CQ-FR, CQ-FR) and the laboratory-susceptible strain (WX-LS) were normalized to RPS7 and RPL49, and the standard deviation is shown on the top of bar. The different letters in AH-FR, CQ-FR, CQ-FR compared with WX-LS indicate significantly different expression determined by t-test (*P*-value < 0.05). The gene *UGT308D3* has significantly different expression in three populations as in RNA-seq analysis

Seven genes significantly upregulated/downregulated in at least two field pyrethroid-resistant populations detected in RNA-seq analysis are subject to RT-qPCR verification using the same sites of samples as in RNA-seq. Most importantly, the *UGT308D3* gene is also significantly upregulated in all three field-resistant populations (Fig. 5). The *UGT308C3* is also significantly upregulated in AH-FR and YN-FR in the RT-qPCR verification, which is the first report of its significant overexpression associated with insecticide resistance. The *UGT302A4* is also significantly upregulated in AN-FR but downregulated in CQ-FR in the RT-qPCR verification. The *UGT308F2* is also significantly upregulated in YN-FR but it is not significantly upregulated in CQ-FR. The *UGT308G2*, *UGT308G3* and *UGT308G4* are also significantly downregulated in CQ-FR and YN-FR in the RT-qPCR analysis.

### Differential SNPs of UGT genes potentially associated with pyrethroid resistance

The SNPs were also screened for all 30 UGT genes and their adjacent regions using re-sequencing technique in order to further identify the genetic basis leading to pyrethroid resistance in *An. sinensis* genome in the present study. This is the first time SNPs of insect UGTs and their adjacent regions especially in terms of insecticide resistance have been investigated.

**Fig. 6:**
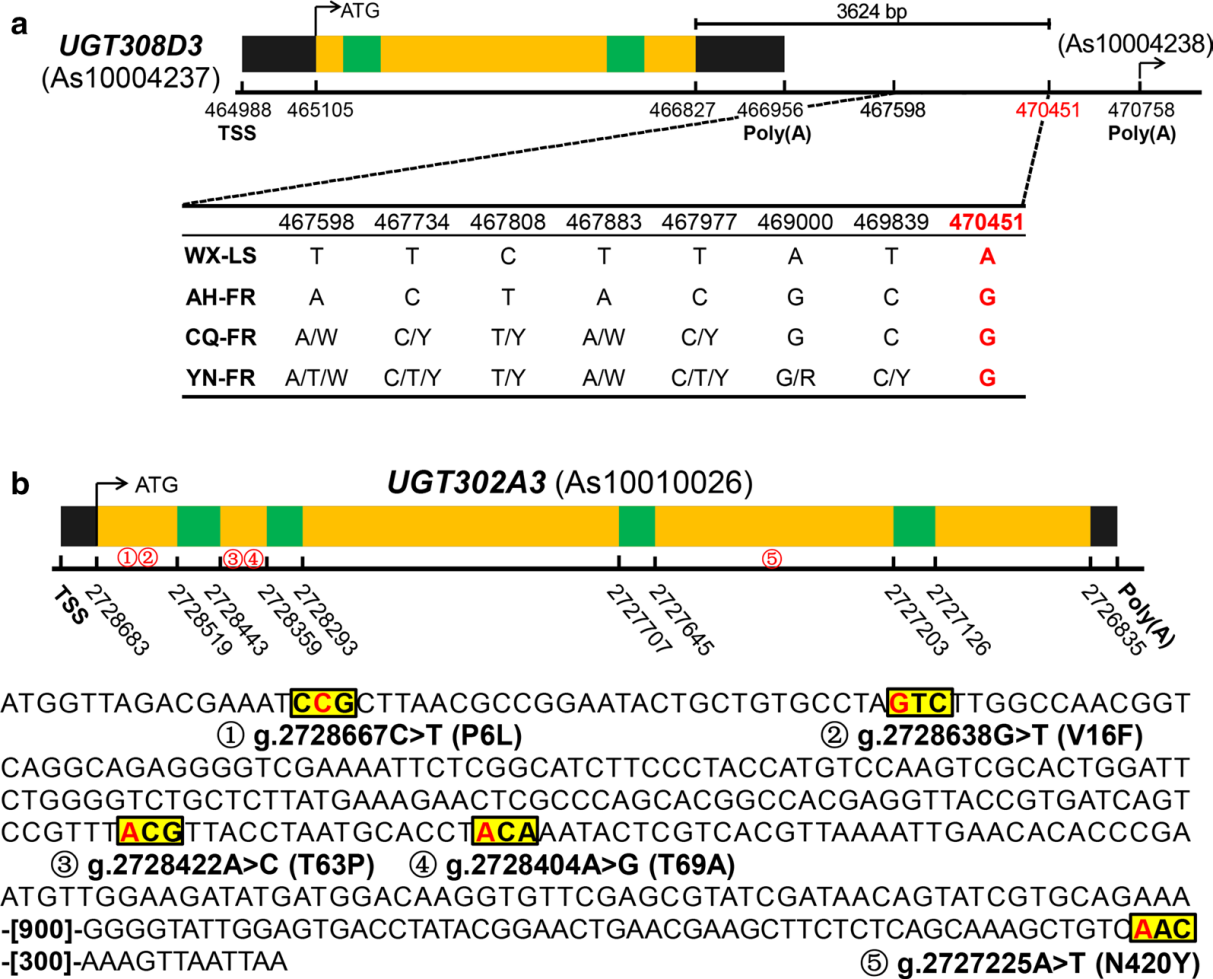
Sequence variation of UGT genes in three populations. In all 30 UGT genes investigated, only the gene *UGT308D3* (**a**) was found to have single nucleotide polymorphism (SNP) variation up- or down-5 Kb intergenic region, only the gene *UGT302A3* (**b**) to have non-synonymous SNP, and no gene to have SNP in intron and untranslated region (UTR). Sites and variations of eight SNPs of *UGT308D3* and five SNPs of *UGT302A3* are shown beneath the illustration of these two genes, respectively. Annotation gene numbers are shown in parentheses, and the black, yellow and green box represent UTR, exon and intron, respectively. TSS: transcriptional start site; Poly(A): poly(A) tail; W: A/T; Y: C/T; R: A/G

The SNP screening of the regulation region of UGT genes exposes a total of eight differential SNPs, and all of them are located in the 3′ flanking region of the *UGT308D3* gene. They are g.467598T>A, g.467734T>C, g.467808C>T, g.467883T>A, g.467977T>C, g.469000A>G, g.469839T>C, and g.470451A>G (Fig. 6a). Out of them, only g.470451A>G is common in three field-resistant populations.

A total of five differential non-synonymous SNPs were identified through the CD sequence comparison between field pyrethroid-resistant and -susceptible populations. All of these five SNPs happen in the *UGT302A3* gene in YN-FR, and they are g.2728667C>T and g.2728638G>T on Exon 1, g.2728422A>C and g.2728404A>G on Exon 2, and g.2727225A>T on Exon 4. The corresponding a.a. substitution of these five SNPs are P6L, V16F, T63P, T69A, and N420Y (Fig. 6b). Four a.a. alterations (P6L, V16F, T63P, T69A) are located in the N-terminal, and one (N420Y) in the C-terminal (Additional file 1).

## Discussion

### Diversity of UGT genes in *Anopheles sinensis*

The number of *An. sinensis* UGTs (30 genes) is comparable with that in *Drosophila melanogaster* (34) and *Aedes aegypti* (32) but greater than that in *An. gambiae* (23). For comparison, the numbers of P450s (112 genes) [45] and CCEs (57) [46] in *An. sinensis* are also larger than those in *An. gambiae* (106 P450s genes and 51 CCEs) [13]. The conservation analysis of protein sequences reveals good conservation of signature motifs and important binding residues, and shows that *An. sinensis* UGT genes have typical characteristic of UGTs as enzymes to catalyze glucosidation. The diversity of *An. sinensis* UGT genes indicates that UGT is a multi-gene superfamily.

### Phylogenetics and evolution of *Anopheles sinensis* UGT genes

In the previous study, the UGT50 family was proposed to be a conserved family with only one member in each holometabolous species [13]. However, two members (*UGT50B4* and *UGT50B5*) are identified in *An. sinensis* genome. Interestingly, the *UGT50B4* might not be able to function as usual UGTs because it is suggested to be a pseudogene due to its lack of TSS. The interspecific conservation in UGT50 suggests a common and essential function for the enzymes in this family, and the function has been discussed in the previous study [13]. The branching pattern of UGT50 mirrors the phylogeny of the four Diptera species investigated [67, 68], and if this family were actually conserved in all holometabolous insects, it may be applied in species phylogenetic analysis as a molecular characteristic.

The gene expansion in UGT308 family likely happened through gene duplication and divergence that increase the diversity of substrates that could be bound for glycosylation and to manage exposure to a changing environment of lipophilic chemicals [1]. Based on previous studies on mosquito UGTs [69, 70], the UGT308 family is speculated to be involved in insecticide resistance. Therefore, the gene expansion in UGT308D and UGT308G may evolve to react to the increasing insecticide stress.

In the present study, 119 UGT genes from the four Diptera species, *An. sinensis* (30 genes), *An. gambiae* (23), *Aedes aegypti* (32) and *Drosophila melanogaster* (34), are divided into 19 traditional families. There is no characteristic a.a. sequence for each of these 19 families to be reported. The work herein does not find any conserved a.a. sequence to identify each of these families either; the herein phylogenetic and evolutionary analyses could not resolve the family classification of the superfamily as well. There is a need for further studies with the addition of more species from different levels of taxonomic taxa to elucidate it.

### RT-qPCR verification of seven UGT genes potentially involved in pyrethroid resistance

Two orthologues of the *AsUGT308D3* in *Ae. aegypti* (*AAEL008560* and *AAEL014371*, i.e., *AaUGT308J1* and *AaUGT308J3* in the present study, respectively) were over-transcribed in insecticide-resistant strains [69, 71]. One orthologue gene of the *AsUGT308D3* in *An. gambiae* (*AGAP006775*, i.e., *AgUGT308D1*) was significantly over-transcribed together with three detoxification enzyme genes in CCE and P450 families [70]. These findings suggest that the *AsUGT308D3* gene could be the essential UGT gene for the participation in biotransformation in the pyrethroid detoxification process.

Three homologues of the *AsUGT302A4* in *Dr. melanogaster* (*DmUGT302C1*, *DmUGT302E1, DmUGT302K1*) showed high amounts in adult midgut [13]. Since the midgut is the major detoxification organs in insects [6, 72, 73], the *AsUGT302A4* may be involved in insecticide detoxification. The *UGT308D3*, *UGT308C3* and *UGT308F2* are shown to derive from recent gene duplication events in herein phylogenetic analysis, and the latter two might also be involved in pyrethroid detoxification, like the *UGT308D3*.

The expression of most UGT genes were significantly repressed after the treatment with two sodium channel blocker insecticides [20]. The role of significantly downregulated genes (*UGT308G2*, *UGT308G3*, *UGT308G4*) in pyrethroid-resistant populations needs further investigation.

In summary, among the seven genes (*UGT308D3*, *UGT308C3*, *UGT302A4*, *UGT308F2*, *UGT308G2*, *UGT308G3*, *UGT308G4*) which are considered to be the main candidates for involvement in pyrethroid resistance, six of them belong to the family UGT308, which suggests that the family is the main family involved in pyrethroid resistance. The family UGT302 is also proposed to be involved in pyrethroid detoxification. Enzyme overproduction can enhance the metabolic capability of detoxification systems and further lead to insecticide resistance [50]. Members of UGTs act as the major phase II enzymes in the detoxification system [24], and have been speculated to be involved in multiple insecticide resistance in the previous studies, such as organophosphorus [28, 29], dichlorodiphenyltrichloroethane (DDT) [30], pyrethroid [31, 36], carbamates [32], neonicotinoids [33–35], and chlorantraniliprole [37]. The present study supports the role of UGT gens in defending against insecticide stress.

### Differential SNPs of UGT genes potentially associated with pyrethroid resistance

Mutations located in flanking region, untranslated region and introns, as *cis*/*trans*-acting elements, can lead to transcription change and further lead to resistance [50, 74]. A large amount of mutations have been reported to be involved in transcription regulation of human UGTs [75–77] and a number of mutations in 3′ flanking region are thought to be involved in regulation of gene expression [78–81]. Since the *UGT308D3* is proposed to be the essential gene associated with pyrethroid resistance due to significant overexpression in all three field-resistant populations, the eight SNPs (g.467598T>A, g.467734T>C, g.467808C>T, g.467883T>A, g.467977T>C, g.469000A>G, g.469839T>C, g.470451A>G) are proposed to be at least partially responsible for the upregulation of *UGT308D3*, especially the mutation g.470451A>G.

Coding sequence alteration can enhance the metabolic capability via deformation of enzyme structure, and further mediate metabolic-based insecticide resistance [50]. A number of researches on human UGTs reveal that the a.a. substitutions at key residues can affect glucuronidation rates and substrate selectivity, and further affect the drug metabolism [82–85]. There is still no report regarding UGT SNPs in insects; however, the non-synonymous SNPs have been reported in P450s [86] and CCEs [87–89], which are thought to be responsible for various insecticides, such as organophosphates, pyrethroid and DDT. Based on *UGT302A3* a.a. sequence analysis, four (P6L, V16F, T63P, T69A) of five a.a. alterations are located in the N-terminal, which is believed to function in binding acceptor molecules [13], and therefore these four a.a. alterations might enhance the catalytic activity of *UGT302A3* and further result in higher insecticide resistance. Phylogenetic analysis in the present study suggests that the *UGT302A3* and *UGT302A4* derive from duplication event. The *UGT302A4* is herein proposed to be involved in pyrethroid resistance via upregulation, while the *UGT302A3* might contribute to pyrethroid resistance via mutations.

## Conclusions

The study reveals the diversity, classification, scaffold location, characteristics, phylogenetics, and evolution of UGT superfamily of genes, and the UGT genes and their mutations associated with pyrethroid resistance in *An. sinensis* genome. There are 30 putative UGTs in *An. sinensis* genome, which are classified into 12 families and further into 23 sub-families. A total of 119 UGTs from *An. sinensis*, *An. gambiae*, *Aedes aegypti,* and *Drosophila melanogaster* genomes are classified into 19 families, of which seven are specific for three mosquito species and seven are specific for *Drosophila melanogaster*. The UGT308 and UGT302 are proposed to main families involved in pyrethroid resistance. The *UGT308D3* is proposed to be the essential UGT gene for the participation in biotransformation in pyrethroid detoxification process, which is possibly regulated by eight SNPs in its 3′ flanking region. The *UGT302A3* is also associated with pyrethroid resistance, and four a.a. mutations in its CDs might enhance its catalytic activity and further result in higher insecticide resistance. This study provides the information frame for UGT superfamily of genes, and lays an important basis for the better understanding and further research on UGT function in defence against insecticide stress.

## Additional file

**Additional file 1.** Multiple alignments of the 11 *Anopheles sinensis* UGTs. Signal peptide in N-terminal predicted by SignalP 4.1 [90] and signature motif are marked with two black horizontal bars above the alignments, respectively. Important catalytic residues, H and D are indicated by red triangles (▼) above the alignment. DBRs in two red square frames refer to donor binding regions with eight important residues interacting with the sugar donor marked (a, b, or c) above the alignments. The negatively charged residue is indicated with a downward arrow. In addition, corresponding amino acid change of the 5 non-synonymous SNPs (➀–➄) detected in the CDs of *UGT302A3* are illustrated above the alignments, with pink boxes indicating the corresponding amino acids on the genome.

## Abbreviations

a.a.: amino acids
aaID: amino acid sequence identity
ANOVA: analysis of variance
BGI: Beijing Genomics Institute
CDs: coding sequence
DBR: donor binding region
DEG: differentially expressed gene
EST: expressed sequence tag
FPKM: fragment per kb per million reads
HMM: Hidden Markov Model
ML: maximum likelihood
RNA-seq: RNA sequencing
RT-qPCR: reverse-transcription quantitative PCR
SNP: single nucleotide polymorphism
TSS: transcriptional start site
UGT: UDP-glycosyltransferase
WHO: World Health Organization
