# Supplementary figures and images for: UDP-glycosyltransferase genes and their association and mutations associated with pyrethroid resistance in Anopheles sinensis (Diptera: Culicidae)
Source: Malar J. 2019 Mar 7;18:62. doi: 10.1186/s12936-019-2705-2 (PMC6407175; doi:10.1186/s12936-019-2705-2)

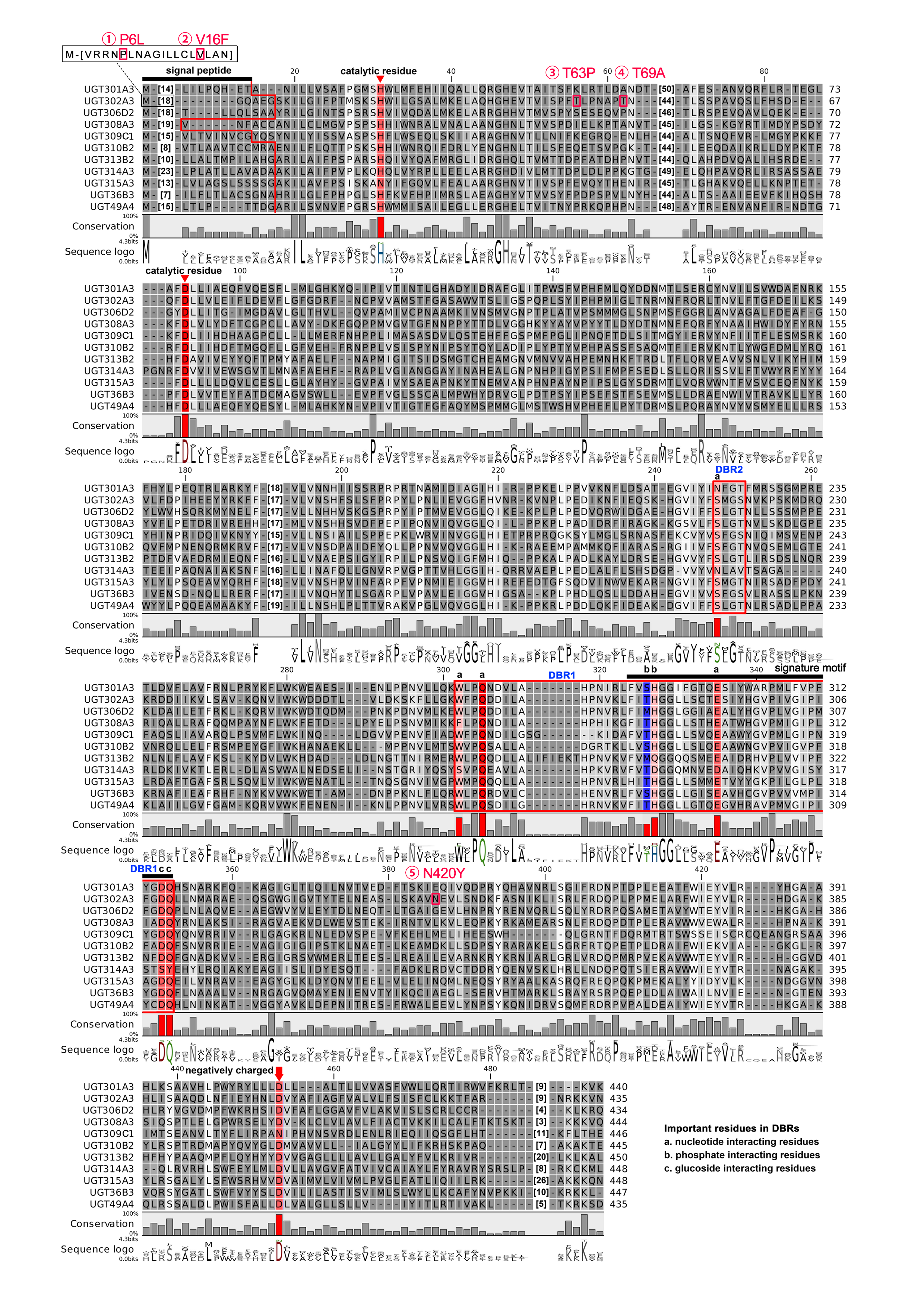

Supplement: Supplementary file 1 — Additional file 1. Multiple alignments of the 11 Anopheles sinensis UGTs. Signal peptide in N-terminal predicted by SignalP 4.1 [90] and signature motif are marked with two black horizontal bars above the alignments, respectively. Important catalytic residues, H and D are indicated by red triangles (▼) above the alignment. DBRs in two red square frames refer to donor binding regions with eight important residues interacting with the sugar donor marked (a, b, or c) above the alignments. The negatively charged residue is indicated with a downward arrow. In addition, corresponding amino acid change of the 5 non-synonymous SNPs (➀–➄) detected in the CDs of UGT302A3 are illustrated above the alignments, with pink boxes indicating the corresponding amino acids on the genome. [file 12936_2019_2705_MOESM1_ESM.tif]
